# Identifying age 26 as a threshold in psychosocial risks associated with child maltreatment among first-time mothers: a cross-sectional study in Japan

**DOI:** 10.3389/fpubh.2026.1747731

**Published:** 2026-04-01

**Authors:** Kaori Baba, Syudo Yamasaki, Junko Niimura, Naomi Nakajima, Daniel Stanyon, Mitsuhiro Miyashita, Satoshi Yamaguchi, Miharu Nakanishi, Sachiko Kita, Mariko Hiraiwa-Hasegawa, Shuntaro Ando, Kiyoto Kasai, Susan M. Sawyer, Atsushi Nishida

**Affiliations:** 1 Unit for Mental Health Promotion, Research Center for Social Science and Medicine, Tokyo Metropolitan Institute of Medical Science, Tokyo, Japan; 2 Graduate School of Nursing Science, St. Luke’s International University, Tokyo, Japan; 3 Department of Psychiatric Nursing, Graduate School of Medicine, Tohoku University, Sendai, Miyagi, Japan; 4 Center for Japanese Studies, University of Michigan, Ann Arbor, MI, United States; 5 Department of Mental Health and Psychiatric Nursing, Institute of Science Tokyo, Tokyo, Japan; 6 School of Advanced Science, SOKENDAI (Graduate University for Advanced Studies), Hayama, Kanagawa, Japan; 7 Department of Neuropsychiatry, Graduate School of Medicine, The University of Tokyo, Tokyo, Japan; 8 The International Research Center for Neurointelligence (WPI-IRCN), The University of Tokyo Institutes for Advanced Study (UTIAS), Tokyo, Japan; 9 Centre for Adolescent Health, Royal Children’s Hospital, and Murdoch Children’s Research Institute, Melbourne, VIC, Australia; 10 Department of Paediatrics, University of Melbourne, Melbourne, VIC, Australia

**Keywords:** child maltreatment, cumulative psychosocial risk, early pregnancy, first-time pregnant women, maternal age, public health, segmented regression, young mothers

## Abstract

**Background:**

Early pregnancy is associated with increased psychosocial vulnerability, which has been linked to factors associated with child maltreatment. However, previous research has predominantly focused on teenagers, and little is known about whether first-time mothers in their early-to-mid twenties also exhibit elevated psychosocial risks. This observational cross-sectional study aimed to determine whether psychosocial risk factors associated with child maltreatment are more prevalent among first-time mothers aged 17–25 years in Japan, compared with those aged 26 years and older.

**Methods:**

Routine administrative data were collected in July 2022 from 429 first-time pregnant women aged 17–43 years who submitted pregnancy notifications to their local municipal office in four municipalities in the Tokyo metropolitan area, Japan. A cumulative psychosocial risk score was calculated by summing six binary indicators: being unmarried, living alone, low household income, low educational attainment, poor mental well-being, and low perceived social support. A segmented regression analysis was used to identify the age at which the association with cumulative psychosocial risk changed.

**Results:**

The association between maternal age and psychosocial risk factors associated with child maltreatment was most prominent up to age 26. A data-driven breakpoint at 25.9 years suggested that younger age was associated with greater psychosocial risk. Younger mothers (ages 17–25, *n* = 158) were more likely to be unmarried, live alone, have a low socioeconomic status, and have lower educational attainment compared with older mothers (ages 26–43, *n* = 271; *p* < 0.005).

**Conclusion:**

First-time pregnant women aged 17–25 years in Japan exhibited higher levels of psychosocial risk factors associated with child maltreatment compared with those aged 26 years and older. These findings suggest that, in addition to teenagers, women in their early-to-mid twenties should be recognized as a priority group for early preventive support within maternal and child health systems. Integrating age-informed approaches into public maternal health policy and antenatal care may help strengthen early prevention child maltreatment.

## Introduction

Early pregnancy is a recognised risk factor for negative health and life outcomes among young women. However, research on adversity during early pregnancy has largely focused on teenagers, rather than women in their twenties (1, 2), who also face economic and social challenges, such as lost educational opportunities (3).

Young adulthood is a distinct period often marked by heightened health and social disparities. According to the Society for Adolescent Health and Medicine’s position statement, individuals aged 18–25 are more likely to experience economic instability, insecure housing, unemployment, and unplanned pregnancy (4). These disadvantages contribute to overall vulnerability and, when present during pregnancy, may increase the intergenerational risk of child maltreatment (5, 6).

In addition to socioeconomic adversity, young pregnant women are also at elevated risk of antenatal depression (7) and limited social support (8), both of which are established predictors of child maltreatment (9). For example, low socioeconomic status, being unmarried, living alone, and lower educational attainment have been independently associated with increased maltreatment risk (10). Moreover, the accumulation of such factors—referred to as “cumulative risk”—has a considerably stronger impact (11).

Psychosocial risk can be considered a multidimensional construct including both socioeconomic disadvantages (e.g., low income, low education, living alone) and psychosocial vulnerabilities (e.g., depressive symptoms, social isolation). This reflects the way these factors often co-occur during early pregnancy and jointly increase maternal vulnerability to child maltreatment. While substantial evidence suggests that psychosocial risks often arise during teenage pregnancy (12), young adults, particularly those in their early twenties also face these challenges beyond the teen years (4). Effective prevention of child maltreatment requires identifying and supporting women at psychosocial risk during pregnancy.

Despite recent definitions of adolescence that extend the age range up to the mid-twenties (3), most guidelines and policies in Japan and other high-income countries continue to define ‘adolescent pregnancy’ as occurring under age 20 and primarily target services at teenage mothers (13, 14). Although there are no national clinical guidelines in Japan explicitly define eligibility criteria for child maltreatment prevention during pregnancy, municipal maternal and child health services often prioritise mothers under 20 based on administrative protocols rather than standardised standards (15). Public health nurses typically assess psychosocial risk at the time of pregnancy notification using standardised tools developed by local governments, initiating interventions such as home visits, welfare referrals, and mental health support when risks such as poverty, isolation,or poor mental health are identified (16).

These risks are not exclusive to teenage mothers. Indeed, first-time mothers in their teens and early twenties are more likely to face multiple, overlapping adversities. The aim is not to exclude older mothers from support, but to ensure that younger mothers, who may be at greater risk, are prioritized for early outreach in resource–limited systems. While most national and local policies continue to prioritise teenage mothers, some maternal and child health initiatives, including home-visiting models in Japan, extend eligibility up to age 25, reflecting a broader understanding of vulnerability in young adulthood (3, 17, 18).

As the age of first pregnancy continues to rise in many high-income countries (18), including Japan (19), psychosocial risks may remain elevated beyond adolescence. Therefore, services and policies should consider this age range within their scope. Yet, limited research has explored psychosocial risk in pregnant women in their twenties, particularly through cumulative risk approaches that allow age–specific comparisons.

We compared psychosocial and socioeconomic risk profiles between younger (17–25 years) and older (26 years and above) first-time mothers to examine whether psychosocial vulnerability remains elevated beyond adolescence and early young adulthood. The age of 26 years was not predefined as a developmental boundary, but used as an analytic comparison group based on prior policy frameworks and observed age-related patterns in psychosocial risks. We hypothesized that cumulative psychosocial risk would decrease with increasing maternal age, while allowing for the possibility that the strength of this association may vary across age ranges during young adulthood.

## Methods

### Study design and participants

This observational cross-sectional study investigated psychosocial and socioeconomic risk factors associated with child maltreatment among first-time pregnant women. The study was conducted as part of a public health initiative commissioned by the Tokyo Metropolitan Government. Participants were recruited from four metropolitan municipalities representing both urban and suburban settings within the Tokyo metropolitan area, Japan. All municipalities operate under the same national maternal and child health framework, including standardized pregnancy notification systems and early risk screening conducted by public health nurses. Although minor variations in service delivery may exist, the overall structure of perinatal public health services is comparable across municipalities.

All first-time pregnant women aged 16 years or older who submitted a pregnancy notification to their local municipal office in July 2022 were eligible. However, no women aged 16 submitted notifications during the study period, so the final age range of participants was 17–43 years. The upper age limit of 43 years reflects the observed age range of first-time pregnant women in the participating municipalities during the recruitment period and does not represent a theoretical or developmental threshold. To ensure adequate representation of younger mothers, we oversampled women aged 17–25 by including those who had submitted pregnancy notifications within the previous 8 months (from November 2021 to July 2022). Oversampling of women aged 17–25 years was conducted to ensure adequate statistical precision for analyses focusing on younger mothers, who represent a smaller proportion of first-time pregnancies but are known to have higher psychosocial vulnerability. Oversampling of specific subgroups is a commonly used strategy in epidemiological research to improve statistical power. To address potential bias arising from recruitment across different calendar dates due to oversampling, sensitivity analyses were conducted adjusting for municipality and the continuous calendar date of pregnancy notification. Eligible women were contacted and informed about the study by public health nurses, and written informed consent was obtained. A histogram illustrating the number of participants at each year of age is provided in Supplementary Figure 1, to demonstrate the full age distribution of the sample.

Participants were excluded at the recruitment stage if they were under the age of 16 or did not provide informed consent. At the analysis stage, participants were excluded from the cumulative psychosocial risk score calculation if data for all six psychosocial risk factors were missing. However, these participants were retained in descriptive comparisons for individual risk factors where relevant.

A total of 429 participants were included in the study. This number reflects all first-time pregnant women aged 17–43 years who submitted a pregnancy notification to one of the four participating municipalities during the recruitment period and who provided written informed consent. No other exclusion criteria were applied.

### Research questions

This study addressed two primary research questions:

1. Can an age-related change point be identified at which the level of psychosocial risk factors associated with child maltreatment shifts among first-time mothers? and.
2. How do individual components of the cumulative psychosocial risk—particularly socioeconomic and psychosocial indicators—differ between mothers who are younger and older than this threshold?

### Data collection

The demographic and psychosocial data included in this study corresponded to the information routinely collected by municipalities as part of standard maternal and child health procedures. However, to ensure consistency across the participating municipalities, all data used in the present study were collected through a standardized, self-administered online questionnaire that was distributed to participants at the time of pregnancy notification. This questionnaire was developed by the research team in collaboration with local public health nurses and was implemented uniformly across all municipalities involved in the study. Participants typically completed the questionnaire at around 14 weeks of gestation (mean = 14.0, SD = 8.0), generally following their initial prenatal interview. In most cases, participants completed the items independently. This time point corresponds to the early second trimester, when pregnancy notification and initial prenatal interviews are routinely conducted in Japan. Assessing psychosocial risk factors at this early stage of pregnancy is consistent with public health practice and allows for the identification of vulnerabilities before substantial pregnancy-related changes occur later in gestation, thereby improving comparability of risk assessment across participants.

### Psychosocial risk factors and cumulative score

In this study, we operationalised “psychosocial risk” as a composite construct that includes both socioeconomic and psychological-social factors. Consistent with recent international frameworks that distinguish between child maltreatment and psychosocial risk factors associated with maltreatment (20), the outcome in this study was defined as cumulative psychosocial risk factors associated with child maltreatment, rather than a direct or validated measure of maltreatment risk. Accordingly, the outcome reflects cumulative psychosocial risk factors associated with child maltreatment. Six psychosocial risk factors were selected based on previous research demonstrating their association with increased risk of child maltreatment. Although other potentially relevant factors (e.g., pregnancy intendedness, employment status, or access to childcare) were considered conceptually during study design, they were not consistently or routinely available across all participating municipalities at the time of data collection. To ensure comparability and feasibility within the context of routine pregnancy notification systems, we therefore focused on indicators that were systematically collected, theoretically grounded, and supported by prior literature.

These factors—unmarried status, living alone, low socioeconomic status, low educational attainment, depressive symptoms, and social isolation—have each been identified in prior studies as independent predictors of perinatal psychosocial adversity and elevated risk of child maltreatment (6–11). In Japan, they are also widely used in routine public health practice as indicators for identifying pregnant women requiring early support.

These included:

1. Marital status: unmarried = 1; married = 0
2. Living situation: living alone = 1; living with partner, parents, or other family = 0
3. Socioeconomic status: annual household income ≤ 2.99 million Japanese yen [JPY] = 1; ≥ 3 million JPY = 0. *Note: At the time of data collection, JPY 3 million was approximately equivalent to USD 21,000*.
4. Educational attainment: junior high or high school = 1; further/higher education = 0
5. Mental health status, assessed via the 5-item version of the World Health Organization Well-Being Index (WHO-5), which is a widely used screening tool for depressive symptoms. Participants rated five items (e.g., “I have felt cheerful and in good spirits”) on a 6-point scale ranging from 0 (at no time) to 5 (all of the time). Total raw scores were converted to a 0–100 scale, with scores <50 indicating possible depression. We used the validated Japanese version of the WHO-5 (21, 22); WHO-5 score < 50 = 1 (depression); ≥ 50 = 0. The WHO-5 is a widely used screening tool for depressive symptoms and reduced well-being in perinatal populations, but it is not intended for diagnostic assessment (21, 22).
6. Social support, measured using a single item from the Japanese version of the Social Support Questionnaire (SSQ), which asks participants how many people they can rely on for support. Responses were scored from 0 to 4 or more people. A score <2 was classified as social isolation. This threshold is based on previous studies using the SSQ in Japanese maternal populations (23, 24); SSQ score < 2 = 1 (social isolation); ≥ 2 = 0. The SSQ, including its short-form components, has been widely used and validated in perinatal populations, including during pregnancy, and brief measures of social support are commonly applied in perinatal and public health research to efficiently identify social isolation in routine settings (25–28).

The six binary variables were summed to produce a cumulative psychosocial risk score ranging from 0 to 6, with higher scores indicating greater psychosocial vulnerability. Consistent with established cumulative risk models, each indicator was assigned equal weight to reflect the additive burden of multiple adversities rather than the relative contribution of individual risk factors, and to enhance interpretability for public health and practice-oriented applications. To avoid bias from missing data, this score was calculated only for participants with complete information on all six items. Participants with missing values were excluded from this analysis but retained in descriptive comparisons for individual risk factors where relevant. In total, 11 participants (2.6%) were excluded from the cumulative score calculation due to missing data.

### Statistical analysis

We performed a descriptive statistical analysis for all study participants. The analyses were conducted to address the two primary research questions described above. To answer the first research question, we conducted segmented regression analysis treating maternal age as a continuous variable. This method estimates potential breakpoints where the association between a continuous predictor and an outcome variable change. The analysis identified a data-driven change point in the association between maternal age and cumulative psychosocial risk, rather than relying on a pre-specified developmental boundary. This empirically identified threshold was therefore used as an analytic reference point to categorise maternal age for subsequent group comparisons. Based on this threshold, we categorised participants (N = 429) into two groups: younger mothers (17–25 years) and older mothers (26–43 years), for analytic comparison of age-related differences in psychosocial vulnerability. To address the second research question, we compared demographic and psychosocial characteristics between the two groups using chi-square tests for categorical variables and t-tests for continuous variables. These two age-based groups served as the primary comparison groups throughout the study to examine age-related differences in individual and cumulative psychosocial risk factors.

To evaluate the robustness of the identified breakpoint and address potential contextual confounding, sensitivity analyses were conducted using segmented regression models adjusted for municipality and calendar date of questionnaire completion. In addition, analyses restricted to participants recruited in July 2022 were performed to assess potential bias related to differential recruitment periods. All analyses were performed using R version 4.4.0 (F Foundation for Statistical Computing, Vienna, Austria).

### Ethics approval

This study was conducted in accordance with the principles of the Declaration of Helsinki. Ethical approval was obtained from the Institutional Review Board of the Tokyo Metropolitan Institute of Medical Science (approval number: 21–39). All participants received written and verbal information about the study from public health nurses prior to participation. Written informed consent was obtained from all participants. For participants aged 17 years, parental or guardian consent was not required in accordance with local ethical guidelines and institutional review board approval. The data used for this study were collected as part of routine municipal procedures during pregnancy notification and prenatal interviews, and no additional procedures were conducted for research purposes.

### Patient and public involvement

Patients and members of the public were not involved in the design, recruitment, data collection, analysis, interpretation, or dissemination of this study. The research was based on secondary use of administrative and routine perinatal care data provided by local municipalities.

## Results

### Sample characteristics

A total of 429 first-time pregnant women aged 17–43 years were included in the analysis. The mean age of participants was 29.0 years (SD = 5.7), and the average gestational age at the time of data collection was 14.0 weeks (SD = 8.0). Overall, 83.9% of participants were married, 96.0% lived with a partner or family member, and 86.6% had a household income of 3 million JPY or more. Full demographic characteristics are presented in Table 1.

**Table 1:** Demographic and psychosocial characteristics of the full study cohort and comparison between younger (17–25 years) and older (26–43 years) first-time mothers.

Characteristic	Mean [SD] or n (%)	Young mothers (<26 years)	Older mothers (≥26 years)	*p*-value^a^
Age (*n* = 429)	29.0 [5.7]	23.2 [1.8]	32.4 [4.3]	<0.001
Marital status (*n* = 429)				
Unmarried	69 (16.1)	**54 (34.2)**	15 (5.5)	**<0.001**
Married	360 (83.9)	104 (65.8)	256 (94.5)	
Living situation (*N* = 423/429)				
Living alone	17 (4.0)	**12 (7.7)**	5 (1.9)	**0.004**
Living with partner, parents, or other family	406 (96.0)	113 (73.4)	256 (95.5)	
Annual household income (Japanese yen) (*N* = 424/429)				
2.99 million or less	57 (13.4)	**43 (27.9)**	14 (5.2)	**<0.001**
3 million or more	367 (86.6)	111 (72.1)	256 (94.8)	
Depression^b^ (*n* = 429)	124 (28.9)	43 (27.2)	81 (29.9)	0.582
Social isolation^c^ (*n* = 429)	27 (6.3)	11 (7.0)	16 (5.9)	0.684
Educational attainment (*n* = 429)				
Junior high school or high school	71 (16.6)	**47 (29.7)**	24 (8.9)	**<0.001**
Further or higher education	358 (83.4)	111 (70.3)	247 (91.1)	

^a^*p* values derived from the chi-square or *t*-test. ^b^Depression was measured using the 5-item version of the World Health Organization Mental Well-Being Index. ^c^Social isolation was measured using one item of the Japanese version of the Social Support Questionnaire. Overall percentages are presented in the total column. Group comparisons were conducted using chi-square or *t*-tests as appropriate. Sample sizes vary due to item-level nonresponse. Bold values indicate statistically significant differences (*p* < 0.05).

### Segmented regression analysis

**Figure 1:**
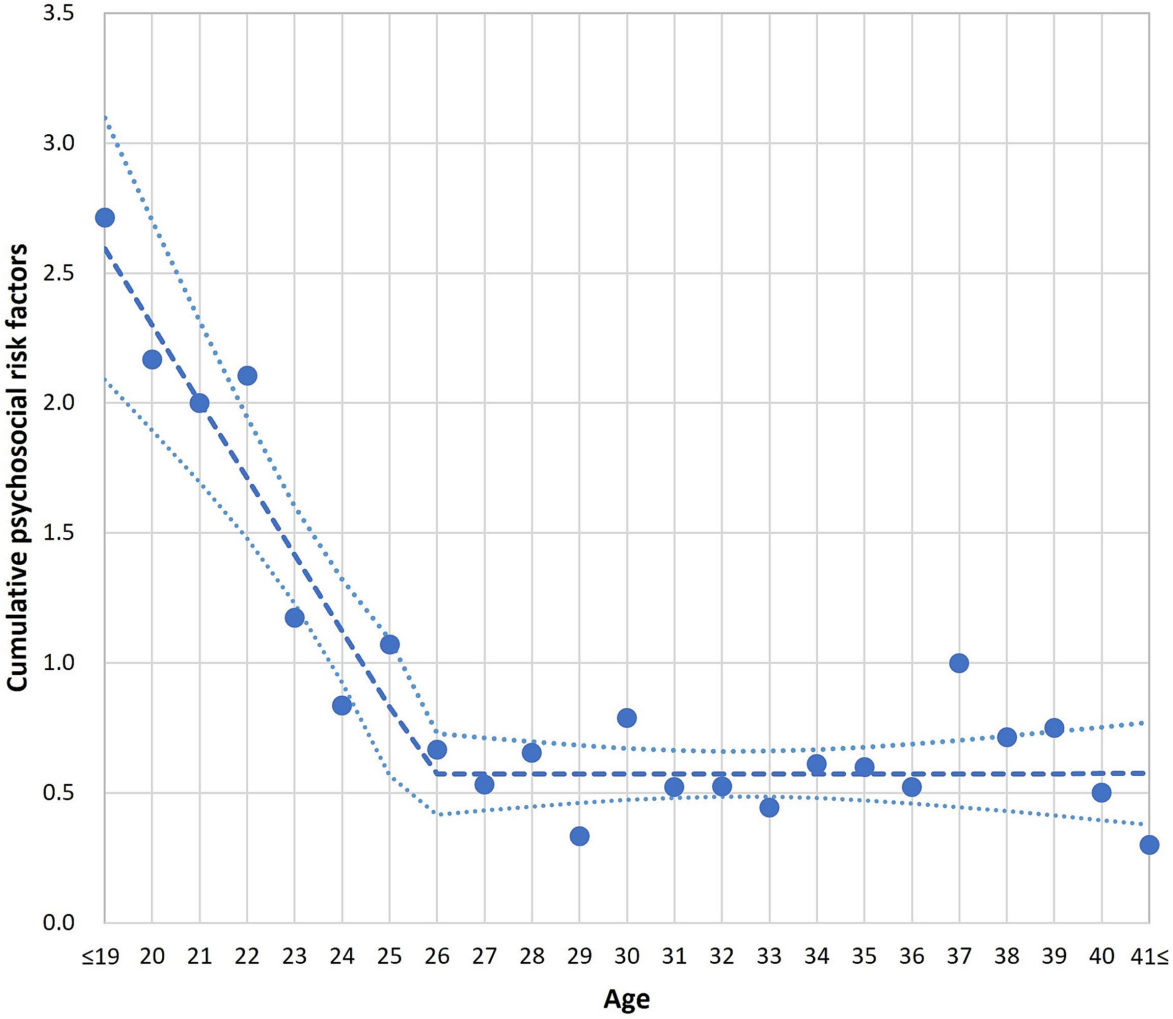
Association between maternal age and cumulative psychosocial risk factors, estimated using segmented regression analysis. Each dot represents the mean cumulative risk score at a given age. The solid line represents the fitted segmented regression line, and the dashed lines indicate the upper and lower 95% confidence intervals. To stabilise the estimation at both extremes of the age range, ages 17–19 years (*n* = 8) were grouped and plotted as ≤19, and ages 41–43 years (*n* = 12) were grouped and plotted as ≥41. See Supplementary Figure 1 for the full age distribution of participants. ≤19 and ≥41 groups were used for visualisation only; all statistical comparisons were conducted between mothers aged 17–25 and 26–43.

In the segmented regression analysis, the breakpoint at which the strength of the association between maternal age and cumulative psychosocial risk factors changed was identified as 25.9 years. This is illustrated in Figure 1, which shows the average number of cumulative psychosocial risk factors score plotted by year of maternal age. Owing to the low number of participants aged 17–19 and 41–43 years, participants at these ages were grouped for analyses. These groups are shown in Figure 1 as ≤19 years and ≥41 years. Specifically, there were only 7 participants (1.6%) aged 17–19 years and 10 participants (2.3%) aged 41–43 years. The full distribution of participant ages is shown in Supplementary Figure 1, which illustrates the number of participants at each age and supports the robustness of the segmented regression findings. The cumulative psychosocial risk score was calculated only for participants with complete data on all six risk indicators (*n* = 418), to avoid bias from missing responses. Based on the segmented regression analysis using this subsample, participants were then categorised into two analytic groups: younger mothers (17–25 years, *n* = 158) and older mothers (26–43 years, *n* = 271). All statistical comparisons presented in Table 1 were conducted using this categorisation.

### Sensitivity analyses

Sensitivity analyses were conducted to evaluate the robustness and stability of the identified age-related breakpoint. When municipality and calendar date (continuous variable) were included as covariates in the segmented regression model, a similar breakpoint was identified at 25.8 years (SE = 0.61). The slope before the breakpoint remained significantly negative (*β* = −0.289, 95% CI − 0.376 to −0.202, *p* < 0.001), indicating a decline in cumulative psychosocial risk with increasing age. In contrast, the slope after the breakpoint was not statistically significant (*β* = 0.002, 95% CI − 0.023 to 0.028), suggesting a plateau in risk levels beyond approximately age 26. Municipality and calendar date were not significantly associated with cumulative psychosocial risk. Analyses restricted to participants recruited in July 2022 showed a comparable age-related pattern, further supporting the stability of the identified breakpoint. Full results of the sensitivity analyses are presented in Supplementary Table 1.

#### Group Comparison by Age Category

Based on the age threshold identified in the segmented regression analysis, we compared individual components of the cumulative psychosocial risk, as well as selected demographic characteristics, between younger and older mothers to aid interpretation of age-related differences. The segmented regression analysis indicated that among participants aged <26 years, the association between maternal age and cumulative psychosocial risk was statistically significant (slope = −0.294, SE = 0.043, *p* < 0.001), suggesting higher cumulative psychosocial risk at younger age. In contrast, among participants aged ≥26 years, the slope was not significant (slope = 0.000, SE = 0.013, *p* = 0.991). These findings support the validity of using 25.9 years as a data-driven threshold to categorise age groups.

Selected demographic characteristics (e.g., marital status, living arrangement, educational attainment, and household income) were included as components of the cumulative psychosocial risk score. These variables are presented descriptively to facilitate interpretation of age-related differences, while the cumulative score represents their combined contribution to psychosocial vulnerability.

Table 1 provides a summary of the full study cohort and presents demographic and psychosocial characteristics compared between younger (17–25 years) and older (26–43 years) first-time mothers. Younger mothers were more likely to be unmarried, live alone, have a low household income (less than 2.99 million yen), and have lower educational attainment (junior high school or high school graduates) (*p* < 0.01). The distribution of cumulative psychosocial risk scores differed markedly between age groups (Supplementary Table 2). Compared with older mothers (≥26 years), younger mothers (<26 years) had a lower proportion of participants with no identified risk factors and a higher proportion with multiple co-occurring risk factors. In particular, 17.2% of younger mothers had three or more risk factors compared with 1.9% of older mothers. These findings suggest that age-related differences in psychosocial vulnerability are driven not only by differences in individual risk factors but also by the clustering of multiple co-occurring adversities among younger mothers, consistent with cumulative risk theory. Supplementary logistic regression analyses demonstrated that younger mothers had significantly higher odds of several socioeconomic risk factors, including unmarried status, low income, low educational attainment, and living alone (Supplementary Table 3). In contrast, depression and social isolation did not differ significantly between age groups. A total of 11 participants (2.6%) were excluded from the cumulative psychosocial risk score analysis due to missing data. Comparisons between included and excluded participants showed no significant differences in maternal age, municipality, or recruitment timing (Supplementary Table 4).

## Discussion

Our findings are consistent with the hypothesis that young adults may be more likely to experience psychosocial risk factors associated with poorer maternal and child health outcomes, including outcomes related to child maltreatment. These risks encompass a range of consequences, including diminished maternal well-being, reduced caregiving capacity, and greater vulnerability to adverse parenting behaviours.

Psychosocial risk factors remained elevated among first-time mothers in their early-to-mid twenties. However, current maternal and child health policies in Japan—particularly municipal home visiting programmes—continue to prioritise mothers under 20 years of age and lack formal mechanisms to extend eligibility to those in their twenties (13–15). Although several home-visiting and family support services (e.g., the Child-Rearing Household Home-Visit Support Program and the Universal Infant Home-Visit Program) may include women in their early twenties in practice, eligibility criteria are not uniformly age-defined, and age-specific evaluations of coverage and effectiveness remain limited. This gap suggests that young adulthood represent a distinct period of vulnerability requiring policy attention. Evidence from this study supports extending the upper age limit of policies and programmes traditionally aimed at pregnant teenagers and teen mothers, to include women in their early-to-mid 20s in high-income countries like Japan. Such a revision aligns with the Society for Adolescent Health and Medicine’s position that adolescent-specific health needs often persist until age 26 (4) and with recent developmental frameworks that define adolescence as spanning from 10 to 24 years (3, 18).

Although the demographic and psychosocial characteristics listed in Table 1—such as being unmarried, living alone, having lower educational attainment, or earning less—may appear unsurprising, their significance lies in their tendency to co-occur among younger mothers. This clustering supports a cumulative risk framework, which posits that the combined burden of multiple moderate risk factors can produce a disproportionate increase in vulnerability to negative outcomes, such as child maltreatment (6, 11). Therefore, even common social characteristics can meaningfully contribute to risk when they overlap rather than occur in isolation.

Given these patterns, young mothers were more likely to be unmarried, live alone, have low socioeconomic status, and report lower educational attainment compared with older mothers. This suggests that interventions should focus on strengthening emotional, social, and financial support for this group. Although we do not advocate for age alone to be used as a screening tool, our findings support its utility as a practical proxy for identifying women who may be at greater psychosocial risk during pregnancy. When comprehensive risk assessment is not feasible in routine care, age could serve as an initial criterion to trigger further evaluation. Age-specific targeting should, however, be situated within a broader risk-informed approach, rather than used independently. In this context, a risk-informed approach refers to the use of readily available information at pregnancy notification, such as basic psychosocial or socioeconomic indicators, to guide further assessment and support. This approach is intended to minimize stigma and reduce the risk of overlooking vulnerable women outside younger age groups by ensuring that age-based identification is supplemented by individual-level risk assessment.

Early identification of psychosocial risk —particularly through age-informed risk screening—may offer a critical opportunity for primary prevention of child maltreatment. Incorporating such age-based screening into antenatal care systems could help ensure timely support for vulnerable women, thereby reducing downstream risks to both mother and child. From a research perspective, priority should be given to intervention studies that test risk-informed home-visiting or case-management models initiated at pregnancy notification, particularly for first-time mothers aged 17–25. In addition, evaluations of multi-component support interventions addressing socioeconomic vulnerabilities, as well as peer-support and relationship-based programmes, are urgently needed to determine which approaches are most effective and feasible within routine municipal health systems. To make early prevention effective, defined responsibilities and cross-sector collaboration are essential. In Japan, public health nurses working in municipal maternal and child health departments are typically responsible for initial risk screening at the time of pregnancy notification. These professionals are well positioned to assess risk factors such as isolation, mental health, and economic hardship. However, effective prevention also depends on collaboration across disciplines, including social workers, perinatal mental health specialists, and community-based services. Developing structured referral pathways and multidisciplinary coordination can help operationalise age-informed prevention models.

In this study, we treated cumulative psychosocial risk as a continuous variable to assess age-related patterns, rather than defining a specific cut-off. While this approach reflects the additive conceptualisation of risk in cumulative risk models (11), future studies could explore whether a particular threshold—such as three or more co-occurring risk factors—is empirically associated with adverse outcomes. Identifying such cut-offs could improve the precision of targeted screening and guide resource allocation within maternal health services.

The breakpoint identified at 25.9 years reflects a change in the pattern of association between maternal age and the cumulative psychosocial risk, rather than a clinical threshold. Although the average cumulative risk score at this breakpoint was below 1.0, segmented regression analysis identifies inflection points in age-related trends rather than absolute levels of risk (29). Psychosocial vulnerability becomes particularly meaningful when multiple (e.g., ≥2) risk factors co-occur (11). Future research should examine how maternal age interacts with such risk clustering to inform screening thresholds and intervention timing. The consistency of findings after adjustment for municipality suggests that the identified age-related pattern is unlikely to be driven by site-specific contextual differences.

It remains uncertain whether these psychosocial risks are concentrated in low-income populations or distributed across socioeconomic strata. Future studies should explore this question to clarify whether targeted or universal interventions are more appropriate. Enhanced social support not only helps to prevent child maltreatment (30), but also improves subjective well-being (31). Although some previous studies have reported higher rates of depression and social isolation among young mothers, our findings did not indicate such patterns during early pregnancy (32), possibly due to the timing of data collection. This pattern is consistent with evidence suggesting that depressive symptoms and social isolation often emerge or intensify in the postpartum period rather than during early pregnancy. For example, postpartum depression affects 10–15% of mothers in high-income countries and is associated with reduced maternal functioning and an increased risk of child maltreatment (33, 34). Similarly, social support frequently declines after childbirth, particularly for first-time mothers, due to isolation and reduced contact with professionals (35). These observations underscore the importance of longitudinal research to examine how antenatal psychosocial vulnerabilities may evolve into postnatal mental health outcomes, and to identify early intervention points that may help prevent escalating risk.

A key strength of this study is that the cumulative psychosocial risk factors analysed are often collected during early pregnancy by standard perinatal services. This implies both high potential benefits and feasibility of using such information to identify at-risk young mothers early, allowing for timely provision of appropriate support (36). To effectively reduce cumulative risk, multifaceted support approaches are needed that address both psychosocial and material vulnerabilities. Investing in social and financial resources for young pregnant women—including access to emotional support, housing assistance, and economic aid—can benefit not only individuals but also next generations (37). For example, conditional cash transfer programmes implemented in many low- and middle-income countries have demonstrated long-term effects in reducing child maltreatment (38, 39). Our findings suggest that similar financial support could benefit young pregnant women in high-income countries. Previous studies on support groups limited to young pregnant women under 25 have shown increased participation rates, suggesting that younger women may be more likely to engage in group-based support interventions (40).

To our knowledge, this is the first study to show that psychosocial risk factors for potential child maltreatment remain elevated up to age 26 among first-time pregnant women. This novel finding extends understanding of vulnerability beyond the teenage years and informs more inclusive maternal health policies.

This study has several strengths. First, it draws on a relatively large community-based sample of first-time mothers in Japan, enabling age-specific comparisons within a population-based context. Second, it leverages standardised, routinely collected psychosocial data, demonstrating the feasibility of using existing maternal health systems for risk screening. Third, the use of segmented regression allows for the identification of a data-driven threshold rather than relying on arbitrary age categories. In addition, the inclusion of multiple municipalities enhances the robustness and generalisability of the findings within metropolitan Japanese settings. The consistency of age-related patterns across municipalities, as demonstrated in sensitivity analyses, suggests that the observed associations are unlikely to be driven by site-specific contextual factors.

However, several limitations should be noted. First, because the study relied exclusively on routinely collected administrative data—obtained at the time of pregnancy notification or prenatal interviews—we were unable to include several important psychosocial risk factors that are well-established predictors of child maltreatment. These include the woman’s own history of childhood abuse (10) (e.g., emotional neglect, physical abuse, sexual abuse), current experiences of intimate partner violence, and partner-related psychosocial or behavioural risks (e.g., mental health problems or substance use), which are not systematically recorded in routine municipal data. The absence of these factors may have led to an underestimation of cumulative psychosocial risk in this study. If included, these exposures could also modify or potentially strengthen the observed age-related risk pattern by capturing additional dimensions of adversity that are not reflected in the current cumulative score. Second, depressive symptoms and social isolation were assessed using brief screening measures. While these tools are widely used and validated for perinatal populations, they may underestimate the complexity and severity of psychosocial difficulties compared with comprehensive diagnostic assessments (21). Third, although the <26 group included a small number of participants aged under 20, the grouping was based on the observed age-risk pattern rather than predefined categories. Future studies could benefit from analysing teenage and early adult mothers separately to clarify their respective risk profiles. Fourth, as a cross-sectional study, our findings cannot establish causal relationships between maternal age and cumulative psychosocial risk, nor can we determine whether these risk profiles predict subsequent child maltreatment outcomes. Future longitudinal studies are warranted to follow mothers from pregnancy through the postpartum period to examine temporal changes in psychosocial risk and to assess downstream outcomes using, where feasible, linkage with home-visiting records and/or administrative or clinical data related to child welfare and maternal–child health. Fifth, reliance on self-reported data may introduce reporting bias. In addition, the cumulative psychosocial risk score assigned equal weight to each indicator and was limited to variables routinely available across participating municipalities. Alternative weighting approaches or inclusion of additional psychosocial indicators (e.g., pregnancy intendedness or employment-related factors) may provide a more nuanced assessment of risk and should be explored in future research. Finally, although our sample was age-diverse, it was drawn from four urban and suburban municipalities in Japan, which may limit the generalizability of our findings to rural areas in Japan or to other high-income countries with different social support systems and policy contexts. Although sensitivity analyses suggested that recruitment timing did not materially influence the observed age-related pattern, the possibility of residual confounding related to differential recruitment periods cannot be completely excluded.

## Conclusion

This study shows that psychosocial risk factors associated with child maltreatment remain elevated among first-time pregnant women up to the age 26 in Japan. These findings highlight the need to recognise not only teenagers but also women in their early-to-mid twenties as a population at heightened psychosocial risk during pregnancy. Incorporating age-informed, risk-sensitive approaches into routine antenatal care may support earlier identification and more responsive support for young mothers who experience psychosocial challenges. Such efforts could contribute to more equitable and preventive maternal and child health policies. Although some key psychosocial factors—such as maternal history of childhood abuse and partner-related risks—were not available in routine data, this study highlights the value of using population-based evidence to inform age-appropriate prevention strategies. Future research should further explore how young maternal age interacts with social and contextual factors to guide tailored interventions.

## Data Availability

The raw data supporting the conclusions of this article will be made available by the authors, without undue reservation.
