# Supplementary material for: Identifying age 26 as a threshold in psychosocial risks associated with child maltreatment among first-time mothers: a cross-sectional study in Japan
Source: Front Public Health. 2026 Apr 1;14:1747731. doi: 10.3389/fpubh.2026.1747731 (PMC13079674; doi:10.3389/fpubh.2026.1747731)
Supplement: Supplementary file 1 [file Table_1.docx]

Supplementary Table 1

Sensitivity analyses of segmented regression models examining age-related changes in cumulative psychosocial risk

| Model | Breakpoint, years (SE) | Slope before breakpoint *β* (95% CI) | Slope after breakpoint *β* (95% CI) |
| --- | --- | --- | --- |
| Primary segmented regression model | 25.9 (0.57) | −0.294 (−0.379 to −0.209) | 0.000 (−0.025 to 0.026) |
| Sensitivity model adjusted for municipality and calendar date | 25.8 (0.61) | −0.289 (−0.376 to −0.202) | 0.002 (−0.023 to 0.028) |

Note:

*β* represents slope coefficients describing the association between maternal age and cumulative psychosocial risk score.

The primary model included maternal age as a segmented continuous variable.

The sensitivity model additionally adjusted for municipality and calendar date of questionnaire completion.

Supplementary Table 2

Distribution of cumulative psychosocial risk scores by age group

| Age group | 0 risk factors, n (%) | 1 risk factor, n (%) | 2 risk factors, n (%) | 3 risk factors, n (%) | 4 risk factors, n (%) | 5 risk factors, n (%) |
| --- | --- | --- | --- | --- | --- | --- |
| <26 years  (n=151) | 51 (33.8%) | 35 (23.2%) | 39 (25.8%) | 18 (11.9%) | 6 (4.0%) | 2 (1.3%) |
| ≥26 years  (n=267) | 143 (53.6%) | 101 (37.8%) | 18 (6.7%) | 4 (1.5%) | 1 (0.4%) | 0 (0.0%) |

Note:

Age groups were defined based on the breakpoint identified in segmented regression analysis.

Percentages are calculated within each age group among participants with complete data for all six psychosocial risk indicators.

The cumulative psychosocial risk score ranges from 0 to 6; however, no participants scored 6 in the present sample.

Supplementary Table 3

Logistic regression analyses of individual psychosocial risk factors by maternal age group

| Psychosocial risk factors | Odds ratio (95% CI) | *p*-value |
| --- | --- | --- |
| Unmarried | 8.85 (4.78–16.39) | <0.001 |
| Low household income | 7.09 (3.72–13.51) | <0.001 |
| Low educational attainment | 4.37 (2.54–7.46) | <0.001 |
| Living alone | 4.41 (1.52–12.82) | 0.006 |
| Social isolation | 1.19 (0.54–2.64) | 0.664 |
| Depression (WHO-5 <50) | 0.88 (0.57–1.36) | 0.556 |

Note:

Odds ratios represent younger mothers (<26 years) relative to older mothers (≥26 years).

Sample sizes vary across analyses due to variable-specific missing data.

Supplementary Table 4

Comparison of participants included and excluded from cumulative psychosocial risk score analysis due to missing data

| Variable | Included (n=418) | Excluded (n=11) | *p*-value |
| --- | --- | --- | --- |
| Maternal age, mean (SD) | 29.07 (5.66) | 25.91 (5.50) | 0.068† |
| Municipality, n (%) |  |  | 0.916‡ |
| Municipality 1 | 67 (15.6) | 4 (0.9) |  |
| Municipality 2 | 209 (48.7) | 2 (0.5) |  |
| Municipality 3 | 91 (21.2) | 3 (0.7) |  |
| Municipality 4 | 51 (11.9) | 2 (0.5) |  |
| Recruitment timing, n (%) |  |  | 0.187§ |
| Early recruitment | 133 (31.0) | 6 (1.4) |  |
| July 2022 recruitment | 285 (66.4) | 5 (1.2) |  |

Note:

SD, standard deviation.

Percentages are column percentages.

† p-value calculated using independent samples t-test.

‡ p-value calculated using chi-square test.

§ p-value calculated using Fisher’s exact test.

Recruitment timing coded as early recruitment (November 2021–June 2022) versus July 2022 recruitment.
